# Exploring the Potential of Therapeutic Agents Targeted towards Mitigating the Events Associated with Amyloid-β Cascade in Alzheimer’s Disease

**DOI:** 10.3390/ijms21207443

**Published:** 2020-10-09

**Authors:** Tapan Behl, Ishnoor Kaur, Ovidiu Fratila, Roxana Brata, Simona Bungau

**Affiliations:** 1 Department of Pharmacology, Chitkara College of Pharmacy, Chitkara University, Punjab 140401, India; ishnoorkaur7@gmail.com; 2 Department of Medical Disciplines, Faculty of Medicine and Pharmacy, University of Oradea, Oradea 410073, Romania; ovidiufr@yahoo.co.uk (O.F.); roxana.gavrila@yahoo.com (R.B.); 3 Department of Pharmacy, Faculty of Medicine and Pharmacy, University of Oradea, Oradea 410028, Romania

**Keywords:** Alzheimer’s disease (AD), amyloid beta (Aβ) cascade, amyloid precursor protein, toxic oligomers, immune response

## Abstract

One of the most commonly occurring neurodegenerative disorders, Alzheimer’s disease (AD), encompasses the loss of cognitive and memory potential, impaired learning, dementia and behavioral defects, and has been prevalent since the 1900s. The accelerating occurrence of AD is expected to reach 65.7 million by 2030. The disease results in neural atrophy and disrupted inter-neuronal connections. Amongst multiple AD pathogenesis hypotheses, the amyloid beta (Aβ) cascade is the most relevant and accepted form of the hypothesis, which suggests that Aβ monomers are formed as a result of the cleavage of amyloid precursor protein (APP), followed by the conversion of these monomers to toxic oligomers, which in turn develop β-sheets, fibrils and plaques. The review targets the events in the amyloid hypothesis and elaborates suitable therapeutic agents that function by hindering the steps of plaque formation and lowering Aβ levels in the brain. The authors discuss treatment possibilities, including the inhibition of β- and γ-secretase-mediated enzymatic cleavage of APP, the immune response generating active immunotherapy and passive immunotherapeutic approaches targeting monoclonal antibodies towards Aβ aggregates, the removal of amyloid aggregates by the activation of enzymatic pathways or the regulation of Aβ circulation, glucagon-like peptide-1 (GLP-1)-mediated curbed accumulation and the neurotoxic potential of Aβ aggregates, bapineuzumab-mediated vascular permeability alterations, statin-mediated Aβ peptide degradation, the potential role of ibuprofen and the significance of natural drugs and dyes in hindering the amyloid cascade events. Thus, the authors aim to highlight the treatment perspective, targeting the amyloid hypothesis, while simultaneously emphasizing the need to conduct further investigations, in order to provide an opportunity to neurologists to develop novel and reliable treatment therapies for the retardation of AD progression.

## 1. Introduction

Amongst neurodegenerative disorders, Alzheimer’s disease (AD) is a chronic, progressive form of the degeneration of neuronal cells (causing brain atrophy) [1], which is considered to be one of the major malfunctions of the central nervous system (CNS). The disease has been prevalent among the general population since the 1900s, when no proper treatment therapy was available to provide a proper cure [2]. In about 60% of AD cases, dementia has been found to be the underlying cause [3]. In the last two decades, AD prominence has drastically increased across the globe, with 36 million cases identified in 2010, and is expected to increase up to 65.7 million by 2030 [2]. Therefore, this has established the need to develop a suitable treatment therapy to retard its increasing prevalence [4]. AD is characterized by severe atrophy of the brain, resulting in a progressive loss of neurons, both in number and mass [3]. The inter-neuronal communication is hampered due to impaired and disrupted electric impulses in the brain, causing dementia and other behavioral alterations [5,6]. This progressive disorder is characterized by gradual elevation in the occurrence of signs and symptoms associated with it, when passing from the initial stage to the next [2]. The majority of AD cases have been reported among individuals of 65 years of age, which creates difficulties for care providers and family members to aid them in routine activities [7]. About 13% of people >65 years of age and 45% of individuals >85 years of age are affected with AD, which is progressively accelerating at an alarming rate [8]. On account of retarded levels of acetylcholine (Ach) in the early stages of AD, the conventional treatment therapies comprise targeting the synthesis and degradation of Ach [2]. Two primary causes responsible for the loss of Ach are either an accelerated degradation rate (due to enhanced acetylcholinesterase levels) or Ach or cholinergic neuron destruction, which results in the loss of cholinergic stimulation, which is essential for behavioral functions [9,10]. Therefore, acetylcholinesterase inhibitors are conventionally recognized as first-line drugs in AD treatment, mainly comprising rivastigmine, galantamine and donepezil, which are commonly employed in mild AD cases [11]. However, after the first 3 months of observed improvement in the condition of AD patients, the action of these compounds is reduced due to the development of tolerance [2]. Additionally, these drug candidates are associated with potential side effects, like diarrhea, vomiting, nausea and abdominal cramps [2]. Furthermore, an N-methyl-D-aspartate (NMDA) antagonist, memantine, is also employed in moderate to severe cases of AD, either individually or as combination therapy with donepezil [2]. However, memantine is also associated with certain side effects, such as headache, mental confusion and dizziness [12]. Therefore, the conventional therapeutic candidates only provide symptomatic relief in AD, without eliminating the actual cause of the disease, and are unable to hinder disease progression at later stages of AD [2]. The accelerating prevalence of AD across the globe and the limited availability of treatment therapies have rendered AD a major health concern.

There are several theories related to AD pathophysiology, among which the amyloid cascade hypothesis is the most relevant and accepted one, which states that the neurologic degeneration occurs as a result of the formation and accumulation of toxic, soluble amyloid beta (Aβ) oligomers, which are formed by the misfolding of Aβ monomers [13]. These monomeric entities are formed by β- and γ-secretase-mediated amyloid precursor protein (APP) cleavage [14], and contribute to the development of larger fibrils and plaque. Studies and investigations conducted in the past have indicated that small oligomers exhibit greater toxicity than Aβ aggregates, where plaques and fibrils are non-toxic, although they are fundamental sources of free amyloid [15].

Certain investigations show that the cell membrane interactions of soluble Aβ oligomers (toxic) contribute significantly to cellular toxicity [15,16], as these oligomeric entities accumulate on the cell surface, resulting in its deformation [15,16,17]. The integrity of the cellular membrane is damaged by the development of transmembrane channels [18,19,20] and pores, which are formed at later stages of AD [21,22]. The amyloid effects on the cell membrane comprise changes in synaptic plasticity, the distribution of receptor proteins and modified signaling processes, followed by the prevalence of more severe malfunctions [16,21]. The Aβ species are located in humans of all age groups and pathologies, however, their exact role is yet to be explored [13]. The Aβ peptides are reported to contribute significantly to the regulation of fundamental neuronal signaling pathways [23] and are thus likely to exhibit neuroprotective functions at low doses [24]. Healthy individuals have been reported to exhibit good clearance potential, facilitating the elimination of amyloid before it attains toxic levels in the brain, therefore minimizing the risk of amyloid-induced cellular toxicity [25]. Therefore, a proper balance between the production and clearance of amyloid peptides exists in healthy individuals [25].

The review targets the amyloid cascade hypothesis of AD and highlights potential treatment therapies relevant to this form of AD hypothesis. It elaborates the beta (β-) and gamma (γ-) secretase modulators, passive and active immunotherapeutic approaches, agents promoting the removal of amyloid aggregates, the peptide inhibitors of amyloid aggregation, natural drugs targeting Aβ peptides and other therapeutic approaches, providing a detailed account of studies and agents associated with the beta amyloid hypothesis. The authors aim to portray the significance of the amyloid cascade as a potential therapeutic target, and the effective results and outcomes shown by various agents in ameliorating AD prevalence, thus providing an opportunity for researchers worldwide to further investigate the role of amyloid peptides in depth and their significance in AD to facilitate the development of a suitable therapeutic regime for AD.

## 2. The Amyloid Beta Cascade Hypothesis

**Figure 1:**
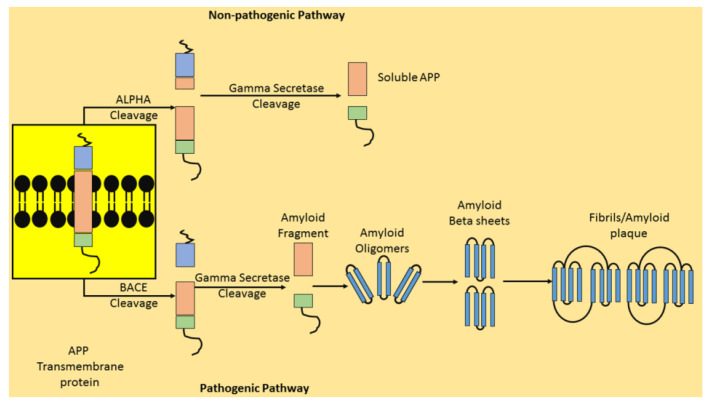
The amyloid cascade hypothesis. BACE—Beta-site APP-cleaving enzyme; APP—Amyloid precursor protein.

The amyloid hypothesis (Figure 1) encompasses a cascade of pathogenic and non-pathogenic events, revolving around the production and aggregation of amyloid beta (Aβ) peptides in the extracellular matrix of brain cells [25].

The Aβ peptide is a fragment of a transmembrane amyloid precursor protein (APP), which is predominantly located in the neuronal and glial cells [26,27]. A sequential cleavage of APP takes place by specific enzymes at different locations, resulting in the production of multiple fragments [2]. Primarily, α-secretase and γ-secretase are the two most significant enzymes which facilitate APP cleavage under physiological conditions, resulting in the production of the APP intracellular domain (AICD) and extracellular soluble fragments, which are non-toxic, enhance synaptic plasticity, regulate neuronal excitability and grant protection from metabolic and oxidative stress [28,29]. This is called the non-pathogenic pathway.

Another group of enzymes, referred to as β-secretase or beta-site APP-cleaving enzymes (BACEs), also induce APP cleavage under disease conditions (pathogenic pathway) [30]. In particular, the β- and γ-secretase-mediated cleavage of APP results in the development of extracellular Aβ42 fragment monomers, which further form loose aggregates of Aβ42 fragments, identified as oligomers (soluble) [28,31,32]. These oligomeric structures further arrange themselves into sheet-like structures, called beta-sheets (β-sheets), which collectively form ordered fibrils, referred to as β-plaques [33,34]. Each strand of the β-sheets is parallel polymerized with an alternate monomer, facilitating the conversion of these sheets into fibrils [2]. The glutamine 15 (Gln15) and glycine 37 (Gly37) components associated with the monomer are found to interact with each other [35]. Plaque deposition occurs on the neuronal cell surface, resulting in deformed cell membranes and altered cell structures [36]. The oligomeric entities are considered to be more toxic than the plaques [37]. The synaptic accumulation of Aβ plaques blocks the neuronal communication, as a result of which the inter-neuronal signal transmission is lost [38]. AD is marked with the occurrence of Aβ plaque [39]. The neuronal immune response stimulates the repeated deposition and accumulation of Aβ plaques, which result in the disruption of brain cells, causing cell death [40]. Notably, a physiological balance is maintained between amyloid development and elimination [41]. The proinflammatory cytokines, reactive oxygen species (ROS), prostaglandins (PGs), nitric oxide synthase (NOS), etc., as well as activated microglia and astroglia, contribute to elevated brain oxidative stress (OS), which in turn causes neuronal cell death [42].

### The Role of Neuroinflammation and Oxidative Stress in Pathophysiology of Alzheimer’s Disease

Alzheimer’s disease is one of the most common neurodegenerative disorders in the world, which encompasses irreversible cognitive defects and potential behavioral changes. The disease is associated with the extracellular deposition of amyloid beta plaques and the intracellular aggregation of neurofibrillary tangles (NFTs). Cytosolic calcium release is promoted by the Aβ42 peptide, resulting in its accumulation in the endoplasmic reticulum (ER), causing elevation in the endogenous levels of glutathione (GSH) and reactive oxygen species (ROS), until a condition of oxidative stress (OS) is induced in the body [43]. Oxidative stress is identified as a primary event in AD pathophysiology and is related to deposition of Aβ plaques, which is further associated with cellular events, like p38 mitogen-activated protein kinase (MAPK) signaling activation, which causes the hyperphosphorylation of tau protein, resulting in the intracellular formation of neurofibrillary tangles (NFTs) and the B-cell lymphoma-2 (Bcl-2)-mediated induction of apoptosis resulting in the mitochondrial release of cytochrome C, as well as T cell infiltration into the neuronal parenchymal cells [44]. On the contrary, systemic or CNS inflammation exerts positive feedback on the over-accumulation of ROS.

Certain studies have reported the elevated response of T helper 17 cells (Th17) in AD. The T lymphocytes from the peripheral blood were evaluated by immunophenotypic and functional parameters in AD patients when the results were compared with patients with mild cognitive impairment and healthy subjects. It was found that the production of Th17-like cytokines, namely, interleukin-6, -21 and -23 (IL-6, IL-21, IL-23), and the expression of retinoic acid receptor (RAR)-related orphan receptor gamma were elevated as a result of in vitro stimulation of naïve lymphocytes from AD patients [43]. The expression of Th2-related transcription factor GATA-3 was significantly elevated only in patients with mild cognitive impairment, which might be a modulatory mechanism to activate Th17 cells. Neuroinflammation occurs as a result of the failure of this mechanism, resulting in AD [45].

The aggregation of misfolded protein in the aged brain causes oxidative and inflammatory harm, resulting in damage to the synaptic connections and energy failure [14]. Elevated nuclear and mitochondrial concentrations of DNA oxidation products are a significant cause of oxidative damage in AD. Additionally, the concentrations of nitrated, glycated and oxidized proteins are found to be increased in helical filaments, plaques and cerebrospinal cord fluid in patients with AD. Advanced glycation end products are reported to be accumulated neurofibrillary tangles (NFTs) and amyloid peptides, resulting in the induction of neurotoxic inflammatory mediators, such as tumor necrosis factor-α (TNF-α), nitric oxide (NO), etc. The impairment of proteosome function occurs as a result of the cross-linking of hyperphosphorylated tau proteins, resulting in the formation of aggregates. The binding of Aβ peptides to transition metals, like copper and iron, initiates a toxic chemical reaction, altering the oxidation states of metals and results in the catalytic production of hydrogen peroxide (H_2_O_2_), which further propagates multiple events, like the Fenton reaction, resulting in the production of toxic hydroxyl radicals and impaired calcium levels. This generates ROS production and excitotoxic response. The soluble oxidized Aβ peptides are accumulated within the synapse, where elevated levels of zinc ions precipitate the copper/iron-metallated Aβ, further developing a reservoir of toxic amyloid peptides [46].

Neuroinflammation significantly contributes to AD progression, which is induced by multiple damage signals, like infection, trauma, tau oligomers, amyloid peptides, oxidative reagents, etc. Neuroinflammation is associated with the unusual release of proinflammatory cytokines, which trigger signaling pathways, aggravating the AD conditions. Besides the neuronal cells, neuroinflammation is also associated with immunological cells, like microglia, astrocytes and peripheral immune cells, resulting in neuroinflammation and neurodegeneration [47].

The interaction between microglia and soluble Aβ oligomers and fibrils via cell surface receptors induces the release of pro-inflammatory cytokines, like IL-1α, IL-8, IL-18, IL-6, IL-23, IL-1β, IL-12, interferon-γ (INF-γ), TNF-α and toll-like receptors (TLRs) [48], chemokines like monocyte chemotactic protein 1 (MCP1), complement proteins, granulocyte macrophage colony-stimulating factor (GM-CSF), prostaglandins, NO, MCP-113, thromboxanes, proteases, leukotrienes (LTs), chemoattractant proteins, pentraxins, protease inhibitors, reactive oxygen species (ROS) and finally activates the nod-like receptor (NLR) family pyrin domain-containing 3 (NLRP3) inflammasome. The accumulation of amyloid peptides and the activation of inflammasomes is mitigated by the genetic amelioration of TLR-4, TLR-6, cluster of differentiation 36 (CD36) or NLRP3, which curb the production of Aβ-induced cytokines. Furthermore, blood–brain barrier (BBB) permeability is elevated, permitting the neuronal entry of leukocytes, followed by the hampering of neurogenesis and upstream activation of nuclear factor kappa-B (NF-κB). Following this, the mitogen-activated protein kinase (MAPK) pathway is activated, with Aβ-dependent pro-inflammatory genetic expression, resulting in the elevation of amyloidogenic pathway events, primarily the synthesis of Aβ peptides [49]. The Aβ-mediated activation of astrocytes not only promotes the atrophy of astroglial cells, but also induces the release of cytokines, interleukins (ILs), NO and other neurotoxic agents. Cognitive potential and synaptic networks are damaged in the case of the loss of astrocytes, which contribute to internalization and degradation of amyloid peptides [50]. A chronic inflammatory state is propagated as a result of the inability to counteract the NFTs and Aβ accumulation. This neuroinflammation affects the crosstalk between neuronal and glial cells, resulting in neuronal death, besides acting in an autocrine manner by spreading reactive gliosis [49].

## 3. Therapeutic Agents Targeting Amyloid Cascade Events

### 3.1. Modulation of Secretase Enzymes

The enzyme β-secretase (BACE) plays a significant role in catalytic actions of APP transmembrane protein [51]. Beta-site amyloid precursor protein-cleaving enzyme (BACE1) complex-mediated cleavage leads to the development of C83 and C99 fragments, which are exposed to γ-secretase, resulting in the formation of extracellular fragments [2]. β-secretase inhibitors are divided into two classes, comprising non-peptidic and peptidomimetic agents. Most of the developmental events account for the selectivity and size of the molecule, where the molecules with larger sizes create issues in CNS drug delivery, due to the presence of the blood–brain barrier (BBB) [2]. Furthermore, selectivity towards BACE1 is also a challenge which needs to be overcome in order to develop potential β-secretase inhibitor agents [2]. A BACE1 isoform, BACE2, is similar to BACE1, however, it is more abundantly present in the pancreas, kidneys, stomach, etc., than in the brain and, therefore, reduced specificity is observed in this case [2]. KMI-429, with a hydroxymethylcarbonyl (HMC) isostere as a component of its structure, was developed and assessed for its BACE1-blocking actions in BACE1-HEK293 cell lines, as well as in wild-type and transgenic (Tg) 2526 mice in vivo [52]. A potential inhibitor of BACE1, N-benzoyloxy-carbonyl-valine-leucine-leucinal (Z-VLL-CHO), was used as a standard reference in the study [2]. A dose-dependent blockage of the formation of soluble APP was revealed by KMI-429 in vitro (IC-50 value—42.8 nM), with 20% retardation of soluble APP levels following intra-hippocampal injection, exhibiting specific results confined to BACE1 only [2]. Additionally, 65.4 + 10.3% and 60.7 + 9.2% reductions in the expression of Aβ42 and Aβ40 in soluble brain fractions at 10mM were reported, without any effect seen on the insoluble components [2]. More effective results of KMI-429 were obtained in wild-type mice, with reductions of 39.8% and 42.9% in the levels of Aβ2 and Aβ40 in the soluble proportions, whereas 31.0% and 34.6% reductions were observed in Aβ42 and Aβ40 levels in insoluble proportions at a 10nmol dose [2]. Due to the structural properties, elevated BBB permeation and low molecular mass of KMI-429, it could show better actions than the standard reference [2]. The therapeutic role of β-secretase inhibitors in the reduction of Aβ is displayed by previous investigations in animal models in which the absence of the β-secretase gene caused only minor alterations in the phenotypic behavior of mice [53].

The first investigational evidence was provided in 2010, when a β-secretase inhibitor, GRL-8234, was employed to treat Tg2576 transgenic mice, and resulted in improved cognitive effects [54]. Moreover, soluble Aβ levels were also found to be retarded in mice [55,56] following GRL-8234 administration [54]. A significant reduction in Aβ plaque load was also observed in aged mice treated with GRL-8234 [57]. BACE1 facilitates APP cleavage, resulting in the formation of Aβ42 and Aβ40 peptide fragments, thus its inhibition shows an appreciable therapeutic strategy for AD [58]. The BACE1 inhibitory actions of iso-liquiritigenin (derived from *Glycyrrhizauralensis*) were evaluated in vitro [59]. Another BACE1 inhibitor is (*R*)-6-[(1,1′-biphenyl)-4-ylmethoxy]-1,2,3,4-tetrahydro-*N*,*N*-dimethyl-2-naphthalene-ethan-amine hydrochloride monohydrate (TAK-070), which is a non-peptidic agent and reduces the expression levels of soluble Aβ, blocks the cerebral accumulation of insoluble Aβ, enhances neurotrophic APP and reduces behavioral problems in AD transgenic mouse models [54]. This agent is considered to be effective and safe, with limited adverse effects due to complete BACE1 blockage [54].

**Figure 2:**
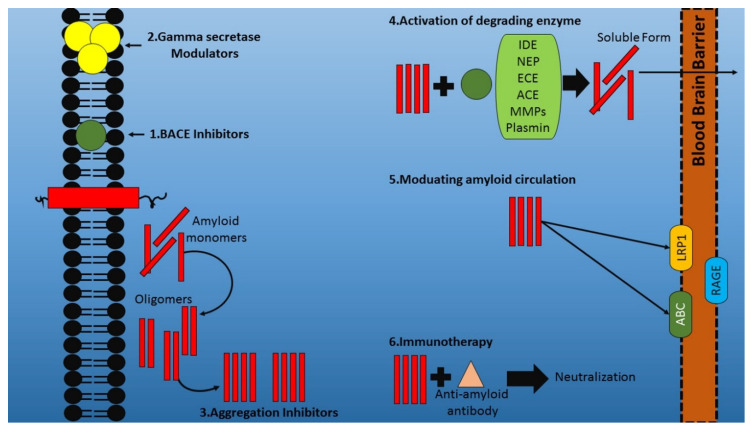
Therapeutical agents targeting the events in the amyloid beta (Aβ) hypothesis. BACE—Beta-site APP-cleaving enzyme.

The γ-secretase complex is developed by linking four subunits together, primarily nicastrin, presenilin, anterior pharynx-defective 1 and presenilin enhancer 2 [60], and is responsible for the cleavage of numerous substrates, including amyloid. It also plays a physiological role in brain development by contributing to neuronal differentiation, as well as regulating a pool of neural progenitor cells [61]. It functions through a notch signaling mechanism and, therefore, notch is recognized as a significant substrate of this complex [62]. The actions of orally administered BIIB042 showed potential results in CF-1 mice, Tg2576 mice (10-month-old and 5-month-old age groups) and F344 rats [63]. The selectivity assessment was carried out on a human neuroglioma cell line (H4 cells), where the Aβ42 expression levels were found to be retarded with a simultaneous elevation in Aβ38 levels, with no significant alterations in the levels of Aβ40 [2]. After the administration of multiple doses of BIIB042 (0, 3, 10, 30 and 100 mg/kg), the concentration of both BIIB042 and Aβ fragments was evaluated [2]. The outcomes demonstrated a dose-dependent decline in Aβ42 fragments and elevation in Aβ38 fragments, which were also confirmed by pharmacokinetic and pharmacodynamic evaluation in other experimental models [2]. Furthermore, BIIB042 portrays an appreciable BBB permeation. Studies were conducted on other γ-secretase modulators, like NGP328 and NGP555 [64], where the latter was more preferred, because of its potential pharmacokinetic characteristics. Moreover, the former candidate exhibited hepatotoxic results at therapeutic doses in rats [65]. The levels of Aβ42 and Aβ0 were significantly retarded by NGP555, simultaneously enhancing Aβ37 and Aβ38 expression levels in male Sprague–Dawley rat models [2]. Daily administration of Tg2576 for 6 months with an NGP555 single oral dose of 25mg/kg was carried out to evaluate alterations in cognitive behavior, which showed a 65% improvement, as assessed by a Y-maze cognition test and further confirmed by a Morris water maze [2]. Combined treatment with a BACE inhibitor and γ-secretase modulator exhibited additive actions in the amelioration of Aβ levels, with limited adverse effects [66]. Additionally, 2-aminothiazole-derived compounds are recognized to be soluble γ-secretase modulators, among which soluble gamma secretase modulator-36 (SGSM-36) was investigated in a Chinese hamster ovary cell line (7PA2) and Tg2576 cells [67]. The levels of Aβ42 were significantly retarded by SGSM-36, with no particular effect on Aβ40 levels, along with an ameliorated Aβ42:Aβ40 ratio [2]. The administration of SGSM-36 (25 mg/kg) once daily for 3 consecutives days was carried out, and plasma concentration of Aβ42 was reduced by 42.0% and 46.2% in TBS-insoluble components and plasma samples [2]. Moreover, the presenilin plaque conformation was changed in cell lines by SGSM-36, resulting in their conversion to a non-pathogenic form [2]. The regulation of the production of Aβ was enabled by blocking the proteolytic processing of APP by β-secretase, followed by γ-secretase, which led to the identification of a therapeutic approach for Aβ-associated diseases [54]. Certain undesired side effects are associated with γ-secretase inhibitors, which question their involvement in inhibiting additional substrates [68]. LY450139, a γ-secretase inhibitor, was evaluated in a phase 2 clinical trial in 2008 and was reported to exhibit adverse effects related to the subcutaneous tissue of the skin [54]. Despite being well tolerated (at doses up to 140 mg/d for 3.5 months) and reducing plasma Aβ levels, a need to further investigate its actions was established [54]. The phase 3 trial of semagacestat (γ-secretase inhibitor) was hindered in 2010, following the worsened cognitive condition and enhanced skin cancer prevalence of drug-receiving patients, unlike the placebo group [54]. Various novel compounds and agents have been developed as γ-secretase modulators, including non-steroidal anti-inflammatory drugs (NSAIDs), which act by shifting their cleavage action from longer to shorter species of β-amyloid peptides, with no effect exerted on the notch cleavage [69]. The toxic actions of γ-secretase inhibitors are exhibited because of their notch cleavage properties [54]. Figure 2 depicts the therapeutic agents targeting Aβ events, including the BACE inhibition and modulation of γ-secretase.

### 3.2. Immunotherapy

Immunotherapy is a disease prevention and treatment strategy which boosts the immune response of the body [2]. Immunotherapeutic approaches are of two significant types, the first one being active immunotherapy and the second one is the passive immunotherapy [2]. Active immunotherapy comprises the incorporation of synthetic Aβ-42 fragments, which in turn activate T cells and B cells, resulting in microglia stimulation and the generation of a cellular and humoral response [70], where the latter is sufficient enough to reduce the damage in AD. The antibodies facilitate the opsonization of Aβ plaques, leading to the activation of B cells and phagocytosis induced by the fragment-crystallizable components of antibodies [71]. Improvement in cognitive potential and the blockage of the aggregation of Aβ peptides was observed when Aβ peptides were administered to AD transgenic mouse models [72]. In the phase 2 clinical trials of AN1792 (the first active immunotherapeutic agent), about 6% of patients suffered from cellular meningoencephalitis [73,74]. Therefore, there was a need for an alternative to target Aβ plaques and hinder their development without the generation of an immune response [2]. Another approach in active immunotherapy comprises the incorporation of Aβ fragments with a carrier protein, resulting in the increased activation of T cells [70]. The Aβ fragments in conjugation with poly-lysine (carrier protein) exhibited potential results in the reduction of AD events in transgenic mice, including retarded levels of neuronal amyloid plaques and the elevated serum expression of amyloid (both bound and non-bound) levels [2]. A study depicted the potential of small Aβ-42 fragments for targeting with no pro-inflammatory response generated [75]. Selective antibodies targeting 4-10 Aβ peptide residues were used, resulting in the blockage of cellular toxicity and the accumulation of Aβ plaques [2]. Thus, antibodies targeting the terminal or central parts of Aβ peptides can render protective effects against the amyloid cascade [2].

Passive immunotherapy comprises methods in which externally produced antibodies are administered, which exhibit limited side effects and target numerous forms of amyloid [76,77]. The development and accumulation of toxic Aβ plaques is prevented by antigen–antibody complexes [2]. Cognitive effects were improved and the expression levels of Aβ peptides were reduced as a result of the administration of antihuman APP therapeutic antibody Fab fragments (NAB61) in transgenic murine AD models [78]. The oligomeric and high-order Aβ peptides comprise linear N-terminal epitopes, which are identified and neutralized by the NAB61 antibody, resulting in improved cognitive and learning potential [2].

The prevalence of the amyloid peptide shift from central to peripheral systems of the body is due to the development of antigen–antibody complexes [2]. Certain molecules are obtained from the genetic engineering of multiple enzymes present in the biological system, like, for instance, a genetically engineered derivative of the peroxisomal antioxidant enzyme catalase (CAT-SLK), a peroxisomal antioxidant enzyme which ameliorated Aβ-associated toxicity in rats [79], and improved long-term memory in rats. Penetration across the BBB is the major challenge that affects the therapeutic efficacy, thus becoming a major concern when incorporating genetically modified antibodies into the neural tissues [2]. About 0.1% of the injected dose is the quantity of anti-amyloid antibodies present at the site [80]. These antibodies are administered via intravenous injection via the intraperitoneal route, at a higher dose so the maximum quantity is unloaded into the blood in the peripheral nervous system [2]. Intracranial or skull cap removal is employed to overcome this problem and retard the expression levels of Aβ plaques within 3 days, however, this generates problems related to surgical methods [2]. A targeted non-invasive technique is therefore employed to facilitate the delivery of these antibodies. The permeability across the BBB to facilitate anti-beta amyloid monoclonal (BAM-10) antibody delivery to the brain by IV administration can be enhanced by the magnetic resonance imaging-guided transcranial focused ultrasound (MRIgFUS) method [81]. MRIgFUS was carried out, trans-cranially, in transgenic TgCRND8 mice, which were treated with MRI and FUS contrast agents, as well as BAM-10 antibody [2].

To facilitate the immunohistochemical detection of BAM-10 after its administration, BAM-10 was biotinylated before incorporation. Biotinylated BAM-10 was found to be present in the target area of right hemisphere, bonded to Aβ plaques, while other areas were devoid of BAM-10 [2]. Therefore, this method successfully facilitated enhanced permeability across the BBB, resulting in BAM-10 delivery [2]. Furthermore, Aβ plaque quantification, following a 4 h of treatment with BAM-10, confirmed the therapeutic potential of the compound [2]. About a 12% mean reduction was reported in treated groups, and no retardation was found in the untreated group [2]. Figure 2 portrays the immunotherapeutic approach for targeting the amyloid beta hypothesis.

Moreover, the Aβ plaques are also found to be associated with complement proteins, which form essential components of amyloid deposits and cerebral vascular amyloids in AD, which are, significantly, found in the initial stages, contributing to the progression of AD-associated dementia, thus establishing the role of a complement system in AD. The short Aβ28 and non-fibrillar Aβ42 peptides can promote the dose-dependent activation of complement component C4, which may take place via contact/kinin system activation, which has been reported to be significantly activated in the cerebrospinal fluid of AD patients [82]. The messenger ribonucleic acid (mRNA) levels in cells for complement C1q and C3 components were monitored using RNA gel blot and non-radioactive in situ hybridization in the frontal cortex of AD patients and control subjects of the same age group [83]. A significant elevation, of about 3.5-fold, was found to occur in the hybridization signal for C1q mRNA, as compared to the control group. Furthermore, it was also reported that there were no variations in C3 mRNA levels between AD patients and the age-matched control group. A close relationship was identified between the transcripts coding for both C3 and C1q and neuronal cells, as observed using radioactive in situ hybridization using digoxigenin-labeled riboprobes [83].

### 3.3. Prevention of Amyloid Aggregation

Despite the enhanced development and aggregation of toxic Aβ peptides, disrupted physiological clearance also plays a significant role in amyloid pathogenesis, particularly in late-onset or familial AD [2]. The ubiquitin–proteasome pathway and lysosome-induced degradation are considered to be the two most significant physiological pathways which facilitate the degradation of peptides in the human body, favoring the clearance of undesirable peptides and proteins [2]. Therefore, the altered expression and functions of these enzyme pathways are considered to be significant factors contributing to AD pathology. The associated enzymes involved are insulin degrading enzyme (IDE), neprilysin (NEP), endothelin-converting enzyme (ECE), plasmin and matrix metalloproteinases (MMPs) (Figure 2) [2]. Enkephalinase is a membrane-bound endopeptidase enzyme which is located in neuronal cells. Elevated levels of residual Aβ peptides are reported in the rat hippocampus as a result of the administration of an NEP inhibitor, thiorphan [2]. NEP expression levels were found to be retarded in AD patients, particularly in the mid-temporal gyrus and hippocampus. Moreover, at presynaptic locations, NEP is considered to promote Aβ clearance, further hindering Aβ pathogenesis [2]. Belonging to the M13 family, ECE is a zinc metalloproteinase which exhibits similarities to NEP, despite the difference that thiorphan does not affect ECE, but the metalloprotease inhibitor phosphoramidon does [84]. A 90% reduction is observed in the amyloid load, outside the cell, in Chinese hamster ovary cells, which was counteracted by phosphoramidon [2]. The overexpression of protein kinase C epsilon type (PKCε) in transgenic mice model retards the levels of Aβ peptide by facilitating the enhanced actions of CNS ECE [85]. Thus, ECE stimulation can be a suitable therapeutic possibility for the clearance of Aβ plaques. Furthermore, plasmin significantly contributes to the degradation of fibrils and non-aggregated monomers, however, its actions are diminished in the brains of AD patients [2]. Lipid rafts (sphingolipid–cholesterol microdomains) are considered to be involved in the production of amyloid fragments. Conformational changes in lipid rafts created a link between the hippocampal levels of plasmin in humans and hippocampal neuronal cells of rats [86]. The serine protease plasmin, confined specifically to the hippocampus, significantly facilitates the degradation of amyloid peptides. The α-secretase-mediated cleavage of APP is preferred in the presence of plasmin [87]. Plasminogen binding to the plasma membrane facilitates the development of plasmin. Thus, the plasminogen activators can aid in regulating levels of plasmin, in order to prevent the development of disease-causing amyloids and favor their clearance [2]. MMPs are pre-propeptides which facilitate breakdown of the extracellular matrix when activated. MMP2, 3 and 9 are the main MMPs which are closely related to the degradation of Aβ peptides. In the presence of Aβ peptides, these MMPs are stimulated [2]. MMP2 and 9 facilitate the direct degradation of soluble Aβ peptides, while MMP3 promotes the activation of other latent forms of MMPs [88]. Synthetic Aβ peptide cleavage is carried out by MMP9, which is latent in the neural tissue of AD patients, which is the reason for amyloid accumulation in AD patients [89]. Similar outcomes were reported in vivo in neuronal tissue in APP/PS1 mice [90]. Furthermore, it was reported that the concentration and actions of the MMP2 and 9 forms of MMPs are elevated by 17β-estradiol (estrogen) at a dose of 10nM [91]. Therefore, the stimulators of MMPs can facilitate the clearance of Aβ peptides.

Different physiological pathways are involved in the elimination of waste products from the body, for instance, astroglial cell-mediated tunnels facilitate the clearance of Aβ peptides, forming a significant component of the glymphatic pathway, which is a fundamental pathway of clearance [2]. Similarly, the blood–brain-associated pathways and meningeal lymphatic pathways are the other systems which facilitate the clearance of Aβ peptides from the cerebral interstitium [2]. Aβ1-42 is the toughest amyloid fragment to be eliminated. The collective functions of all the elimination pathways play a significant role in the clearance of Aβ peptides [92]. The glymphatic system promotes the elimination of Aβ peptides (soluble) from the interstitium. In aged mice, the pathway is reduced by 40%, which poses a significant risk for the development of late-onset AD [93]. The size of Aβ peptide fragments and the availability and activation of aquaporin-4 (AQP4), along with sleep and arterial pulse rate, are the essential factors which play a significant role in regulating the actions of the glymphatic system. Glymphatic stasis is marked by the aggregation of Aβ plaques in the periarterial space. During traumatic brain injury associated with AD, AQP4 mislocalization occurs as a result of perivascular inflammation and, therefore, influences glymphatic clearance [93]. The Aβ clearance by accelerated interstitial fluid (ISF) bulk flow towards the cerebrospinal fluid (CSF) accounts for about 40% (approximately), whereas the remaining 60% is facilitated by the blood–brain barrier transport system [2]. Two major efflux transporters, ATP-binding cassette transporters (ABC transporters) and low-density lipoprotein (LDL) receptor-related protein 1 (LRP1), block the accumulation of Aβ peptides in the interstitium and form the important segments of the blood–brain barrier transport pathway [2]. Simultaneously, a receptor for the advanced glycation end-product (RAGE) influx transporter promoted the reverse transportation of circular Aβ to the interstitium [2]. The circulating Aβ peptides form a complex with multiple agents, like the soluble form of RAGE (sRAGE), serum amyloid P (SAP), anti-Aβ immunoglobulin G (IgG) and the soluble form of LRP (sLRP), hindering their interaction with the RAGE transporter, thereby reducing the entry of Aβ peptides into the interstitium and driving them towards systemic elimination [93].

#### Peptide Inhibitors of Amyloid Aggregation

The amyloid peptide fragment KLVFF was revealed to be an aggregation inhibitor in 1996, which led to the identification of the significance of peptide-based ligands, developed from the amyloid sequence itself [94]. The reduction in fibrillization reveals the utility of these peptides as ligands, with the potential to influence fibril formation dynamics [13]. In this study, 31 decamer Aβ peptides were tested, followed by the identification of decapeptides with the greatest affinity. These were then truncated and mutated to identify the minimum sequence required to inhibit the interaction. Finally, the pentapeptide KLVFF, i.e., Aβ16-20, was identified [94]. The sequence KLXXF was recognized to be significant for Aβ peptide binding [13]. Furthermore, the toxicity of Aβ peptides was found to be reduced by Aβ15-25, linked to repeated oligolysine (disrupted element) [13]. The peptide inhibitor (PI) induced alterations in the kinetics of aggregation and higher-order conformational changes in fibrils by reducing the fibril length and enhancing the entanglement [95]. However, this peptide inhibitor failed to inhibit the block β-sheet binding and formation of fibrils. Thus, this portrays the significance and influence of aggregated Aβ structures in cellular toxicity. The identification sequence KLVFF was modified by the addition of repeating oligoproline units [13]. The binding of β-sheets is impaired by the ring structure of proline side chains, alongside a reduction of amyloid stacking capability to grow into larger fibrils via a dynamic competitive process [96]. Moreover, the production of amyloid peptide fibrils was blocked by KLVFF amino acid sequence shuffling, which possessed similar binding characteristics. Therefore, the amyloid ligand hydrophobicity was found to be essential for effective amyloid binding [96]. Thus, these initial preliminary investigations have created a pathway for the advanced design of the subsequent generation of peptide inhibitors.

Different characteristics form the development criteria of amyloid aggregation inhibiting agents, such as binding affinity, immune system evasion and stability, as well as permeability across cell membranes and the blood–brain barrier [13]. Designed from the KLVFF sequence, OR2 (peptide inhibitor) comprises a terminal charged amino acid residue and a glycine spacer [13]. The aqueous solubility was improved, and the formation of fibrils was impaired by these charged terminal residues [13]. Furthermore, early Aβ aggregation was regulated by OR2, which also facilitated the protection of SHSY-5Y cells from cellular toxicity induced by amyloid peptides [97]. The amino acids were replaced with their respective D-enantiomer in order to enhance proteolytic stability and reduce the immune response [13]. However, a single replacement is be enough for the regulation of the biological actions of the original peptide, as peptide binding reversal is also required. The “retro inverso” form of the peptide (RI-OR2) effectively blocked oligomerization and enhanced the survival rate of SH-SY5Y cells against the toxicity of Aβ peptides, and also occurred in blood serum in humans and neuronal extracts in a stable form for at least 24 h [98]. The designing and screening of therapeutic drugs and proteins, which interact with and hinder oligomerization, via computer-aided drug design (CADD), are recognized as a rapid and economical methods for the development and screening of therapeutic agents [99]. The design of the drug for blocking amyloid oligomerization starts with a basic lipid-loving (lipophilic) amyloid recognition sequence: KLVFF, KVLFFAE and LVFFAE [94]. Numerous amino acids located in the sequence are N-methylated in order to prevent them from favoring Aβ oligomer growth and exhibiting permeability across biological membranes [100]. Different forms of substitutions and structural changes are made to the peptide, namely γ-diaminobutyric acid addition as an N-terminal residue, to facilitate enhanced binding potential with amino acid D_23_ and the replacement of lysine with ornithine, a synthetic amino acid which enhances electrostatic side chain binding potential with E_22_ [99]. The replacement of various lipophilic residues optimizes the hydrophobic binding potential between the drug candidate and amyloid peptide target. The lipophilic aromatic amino acids can possibly also be replaced, as these acids are considered to be significant for the identification of peptides and proteins, including amyloids [101]. With this series of inhibitors, the major agents (depending upon molecular dynamics simulations) were analyzed for their anti-aggregatory actions by using western blot assays, thioflavin T fluorescence assays and circular dichroism [99]. The success of molecular dynamics (MD) simulation and thioflavin T fluorescence assays were found to be closely related, portraying the success of in silica drug screening and design [13]. These inhibitors are able to ameliorate interactions between single amyloid monomers, as confirmed by using atomic force spectroscopy [102]. At present, different inhibitors with varying modifications, the administration of D-amino acid residues and numerous predicted binding orientations are being tested and screened both via in vitro assays and direct force measurements [13].

Furthermore, peptide inhibitors (PIs) serve as an appreciable preventive technique for monoclonal antibodies (MAb), because of their economical production, versatility, smaller sizes and safety portfolio [13]. The MAbs and antibody fragments engineered by taming fragments and crystallizable (Fc) regions are considered to be safer substitutes for traditional PIs, however, they do not exhibit appreciable advantages over peptide inhibitors [13]. To enhance the permeability across the BBB, the peptide inhibitors are easily altered by adding shuttling molecular agents and targeting agents [13]. A cell-penetrating peptide (CPP) was isolated from human immunodeficiency virus (HIV) regulatory protein and was used to modify a peptide inhibitor which potentiated the cellular and neuronal delivery of the peptide, with improved characteristics in a transgenic mouse model [103]. Furthermore, MAbs can also be enhanced by inducing bi-specificity with the ability to interact with amyloid peptides and certain characteristics of the BBB, for an enhanced drug delivery paradigm [104,105]. In AD prevention, natural clearance is provided by the blockage of the aggregation of Aβ peptides, where the peptide inhibitors play a significant role.

### 3.4. Autacoid Local Injury Antagonist Amides (ALIAmides) as a Novel Therapeutic Strategy in AD

Autacoid local injury antagonist amides (ALIAmides) refer to the group of endogenous bioactive lipids, comprising palmitoyl ethanol amide (PEA), which play a significant role in the processes of inflammation, pain and lipid metabolism in the body. These compounds mediate the downregulation of mast cell activation, due to which they exert anti-inflammatory and anti-hyperalgesia effects [106]. A lipid mediator-producing program of resolution counteracts the chronic inflammatory responses, resulting in the amelioration of inflammation, which is a central contributor to neurodegenerative disorders. These lipid-signaling molecules (ALIAmides) include N-arachidonoyl ethanolamine and its congener (PEA), as well as N-acylethanolamines. PEA is responsible for maintaining cellular homeostasis in the presence of external stress, like inflammation, and has exhibited effective results in mast cell-mediated models of neurogenic inflammation and neuropathic pain [107]. The ultra-micronized/micronized form of PEA exhibits greater oral efficiency, unlike naive PEA, in inflammatory pain models. The co-ultra-micronized form of PEA, in combination with luteolin (flavonoid), exhibits better potential results than PEA alone, which is devoid of antioxidant properties [107,108]. Therefore, PEA can serve as a suitable therapeutic target to ameliorate the inflammatory responses associated with neurodegenerative disorders like AD.

A study demonstrated the neuroprotective effects of oxazoline of PEA (PEA-OXA) in spinal cord injury (SCI)-induced and traumatic brain injury (TBI)-induced secondary neuroinflammation in mice models, where 10 mg/kg of PEA-OXA was administered intraperitoneally and orally, 1 and 6 h after trauma, resulting in mitigated histological alterations as a result of trauma induction and curbed motor and behavioral defects. Furthermore, the level of neurotrophic factors, like brain-derived neurotrophic factor (BDNF), glial cell line-derived neurotrophic factor (GDNF) and neurotropin-3, was found to be elevated by PEA-OXA therapy, along with reduced expression of glial fibrillary acidic protein, NF-κB and IKB degradation and curbed expression levels of pro-inflammatory mediators, such as cyclooxygenase-2 (COX-2), tumor necrosis factor-α (TNF-α), inducible nitric oxide synthase (iNOS) and IL-1β. N-acylethanolamine hydrolyzing acid amidase (NAAA) serves as a significant alternative therapeutic strategy in the management of neuroinflammation, which is modulated by PEA-OXA treatment [109]. Amyloid peptide-induced astrogliosis can aggravate AD etiopathogenesis by promoting the release of pro-inflammatory and pro-oxidant mediators. Therefore, AD progression cam be controlled by promoting the release of pro-angiogenic factors during astrogliosis. A study demonstrated the effects of PEA in angiogenesis and neuroinflammation associated with AD, by using Aβ-treated and untreated C6 rat astroglioma and human umbilical vein endothelial cells (HUVECs). PEA was found to alleviate the nuclear levels of MAPK1 and vascular endothelial growth factor (VEGF) in the cytoplasm of C6-conditioned HUVECs, thus establishing the clinical utility of PEA in AD [110].

### 3.5. Other Agents Targeting Aβ Deposits

An active immunotherapy clinical investigation, conducted in 2003 with AN1792 (a full length Aβ peptide), was discontinued after meningoencephalitis was reported to occur in 6% of the patients, which caused neurological and cognitive impairment in some patients [111]. The response of the antibody towards AN1792 was specifically directed towards the N-terminal residues, as evidently indicated by subsequent epitope mapping [71]. Numerous studies in mouse models have shown that Aβ N-terminal-targeted antibodies, without T cell-activating epitopes, inhibit neuronal toxicity and fibrillogenesis [75]. Bapineuzumab is a monoclonal antibody (entirely humanized), which exhibits specificity towards the Aβ N-terminal region [54]. MRI-related abnormalities were reported on fluid-attenuated inversion recovery (FLAIR) sequences in phase 1 clinical trials [54]. These changes were found to be resolved within the following weeks after the magnetic resonance imaging (MRI) scans were performed repeatedly. Other investigations repeated the occurrence of vasogenic edema because of changes in the vascular permeability due to bapineuzumab binding with Aβ in the walls of blood vessels [54]. Vasogenic edema is also associated with microhemorrhage. Furthermore, the lower doses of bapineuzumab exhibited better Mini-Mental State Examination scores, unlike placebo [54]. However, similar results were not observed in case of higher doses, which was primarily associated with MRI FLAIR abnormalities [112]. Numerous biochemical and clinical characteristics, such as disrupted insulin signaling, are found to be common to diabetes and AD, where the former is considered to be a risk factor for the latter [113]. The desensitization of insulin receptors is among the various other defects in AD, which is referred to as type 3 diabetes [114]. Therefore, type 2 diabetes can be a therapeutic target in the development of an effective treatment therapy for AD. The stimulation of glucagon-like peptide (GLP-1) in animal and cellular AD models retards the accumulation and neuronal toxicity of Aβ peptides [115]. Other investigations depict the neuroprotective and APP-retarding potential of GLP-1, as well as its role in ameliorating the levels of Aβ peptides in wild-type mice [116]. All these outcomes contributed to the preclinical data for translational investigations in diabetic and/or early AD patients [115]. An inverse relationship was found between the use of statins and the risk of the development of AD when the results were compared to the patients who never received these cholesterol-lowering drugs [117]. The lipophilicity of statins had no involvement in the protective actions exhibited by them. However, certain investigations have also reported the worsening of cognitive potential in patients administered with statins [54]. Statins were reported to stimulate the microglia to release insulin-degrading enzyme, resulting in the degradation of Aβ peptides contained outside the cells [54]. The level of insulin-degrading enzyme in the blood serum was elevated as a result of treatment with statins, while it was retarded in the cell pellets, which showed the selective stimulation of the secretion of insulin-degrading enzyme in the peripheral cells [54]. The mice with depleted levels of microglia exhibited enhanced levels of soluble Aβ, which shows the involvement of microglial cells in Aβ metabolism [118].

**Figure 3:**
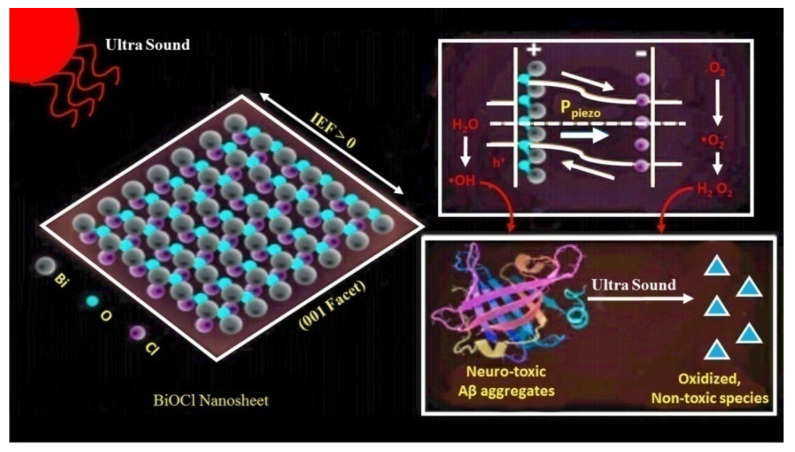
Piezoelectric breakdown of Aβ aggregates on the surface of bismuth oxychloride (BiOCl) nanosheets; BiOCl—bismuth oxychloride.

Furthermore, NSAIDs were reported to provide long-term benefits in AD patients, where the clearest results were established in the case of ibuprofen [119]. The intraneuronal oligomeric Aβ levels were reported to be reduced and cognitive potential was improved in young 3× Tg-AD mice administered with ibuprofen [54]. This drug candidate was considered to act in an enantiomer-specific manner to block the activation of nicotinamide adenine dinucleotide phosphate oxidase and the development of reactive oxygen species (ROS) [54]. This is linked to a curbed level of Aβ deposits and retarded oxidative stress in murine AD models. However, data derived from mice models show that ibuprofen acts via multiple independent pathways to influence the pathologic events related to AD [54]. Other drugs of this category, like celecoxib [120] and naproxen, were not reported to improve cognitive potential, as per the Alzheimer’s Disease Anti-Inflammatory Prevention Trial (ADAPT) [121]. However, NSAIDs are still considered to be potential therapeutic agents, as they are capable of modulating the effects of γ-secretase enzyme [54]. Moreover, certain substances, referred to as piezoelectric materials, facilitate the transference of charge carriers to reactants when they receive mechanical stimuli [122], resulting in the induction of electrochemical reactions [123]. A study evaluated the activity of piezoelectric bismuth oxychloride (BiOCl) nanosheets in promoting the breakdown of Aβ aggregates via ultrasound-induced redox reactions [123]. Thus, these BiOCl nanosheets are biologically compatible materials which exhibit piezoelectric actions in response to ultrasound [123]. It is well known that sonic-activated BiOCl nanosheets induce oxidative stress, promoting the destabilization of β-sheets. Additionally, these nanosheets have been observed to minimize the neurotoxic potential of Aβ aggregates (Figure 3) [123].

### 3.6. Natural Drugs Targeting Amyloids in AD

The growing herbal and phytoconstituent industries have exerted numerous potential benefits in various forms of disorders. Natural compounds comprising dyes and drugs can exhibit significant responses in neurologic disorders like AD. The oldest dye used in the histological staining of Aβ deposits is Congo red [124], which inhibits amyloid formation at high doses [125]. However, its toxic effects minimize its therapeutic abilities. Other amyloid-inhibiting dyes include methylene blue [126,127], thioflavin T [128], orcein [129] and curcumin [130].

Methylene blue is a phenothiazine compound, with BBB permeability and high bioavailability characteristics [131] and exhibits numerous pharmacological benefits as an anti-inflammatory agent, urinary antiseptic and treatment of methemoglobinemia [132]. Methylene blue was reported to enhance the memory and learning potential in 3 × Tg AD mice, along with the retardation of soluble Aβ deposits, primarily due to enhanced proteosome function. A phenoxazine dye, orcein, is derived from *Roccella tinctoria*, and promotes the formation of fibrils of Aβ peptides and reduces the levels of oligomeric and protofibrillar forms of peptides.

The polycyclic polyphenols constitute another category, comprising antioxidant compounds, like resveratrol [71], *Camilla sinensis* (tea plant)-derived catechins, curcumin and dopamine [72]. Epi-gallocatechin-3-gallate (EGCG) is the major constituent of *Camilla sinensis,* which directly interacts with a large quantity of proteins (which contribute to protein misfolding disorders), promoting the inhibition of their fibrillization and the development of stable, spherical aggregates [133]. These aggregates do not exhibit a cellular toxicity profile, having a lower β-sheet content, unlike fibrils, and do not catalyze the formation of fibrils [133]. Furthermore, silibinin (a flavonoid), which is derived from *Silybum marianum*, exhibits a dual inhibitory effect, blocking the actions of acetylcholinesterase and Aβ peptide aggregation [134]. This compound was reported to ameliorate Aβ aggregation in APP/PS1 transgenic mice, as evidently confirmed by circular dichroism (CD) and transmission electron microscopy (TEM) [134].

In a study, docking and atom molecular dynamics simulation was employed to evaluate the interaction of β-sheets and repetitive units of proline, i.e., a β-sheet breaker [135]. Proline was reported to break the amyloid protofibrils, resulting in the breakage of the β-sheet structure [135]. Some cases reported that this compound promoted the production of 3_10_ helices, which further contributed to the unfolding of the β-sheet structure [135]. Furthermore, proline also impaired the hydrogen bonds and salt bridges between the chains, along with loosening the tight interatomic arrangement of atoms. Additionally, proline possesses the ability to interact with the charged residues [135].

**Figure 4:**
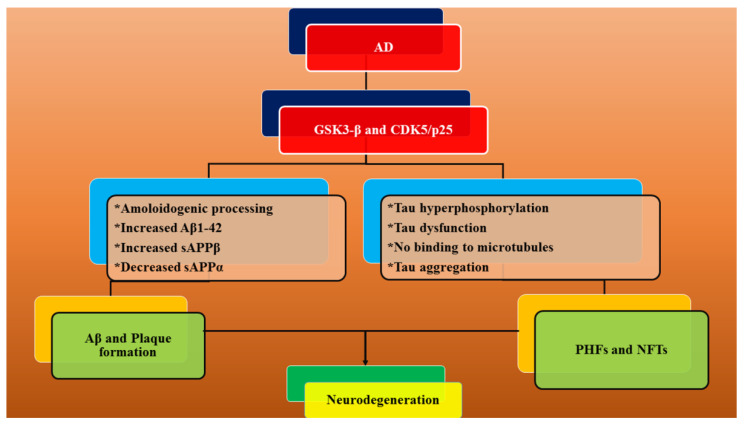
Flavonoid-mediated inhibition of glycogen synthase kinase-3β (GSK-3β) and cyclin-dependent kinase-5 (CDK-5), which facilitates the blockage of Aβ aggregation, amyloid precursor protein (APP) alterations and tau phosphorylation. NFTs—Neurofibrillary tangles.

Moreover, NeuroDefend (ND) is a Chinese medicine formulation which has been reported to ameliorate Aβ and tau pathology in transgenic mice models [136]. It has exhibited improved cognitive and memory functions in 3×Tg-AD and 5×familial AD (FAD) mice [136]. ND retarded the APP levels, Aβ- and 4G8-positive amyloid loads and APP-C terminal fragments in 3×Tg-AD mice, therefore exhibiting effective benefits in AD patients [136]. Naturally present dietary flavonoids exhibit protection against AD by blocking the conversion of Aβ monomers into neurotoxic oligomers [137,138]. These agents inhibit the activation of cyclin-dependent kinase-5 (CDK-5) and glycogen synthase kinase-3β (GSK-3β), as well as modulate the secretase enzymes, resulting in the hampering of Aβ aggregation, APP alterations and abnormal tau phosphorylation (Figure 4) [137]. Table 1 summarizes the therapeutic agents targeting Aβ peptides in AD.

## 4. Future Prospects and Conclusions

The permeability of therapeutic agents across the BBB is the major challenge associated with AD, which can nowadays be overcome by nanotechnological approaches which are based on nanoparticles comprising a nanocore structure, an Aβ-targeting ligand and certain surface alterations to enhance permeability across the BBB [13]. The specificity of the resting motor threshold (RMT) and high capacity utilization of the active motor threshold (AMT) contribute significantly to the effective neuronal delivery of compounds and aid in the improvement of nanoparticle surface characteristics [13]. Furthermore, a detailed understanding of the exact pathogenesis of AD would be helpful in determining the therapeutic approaches for the disorder. Currently, a number of AD hypotheses have been proposed, among which the amyloid cascade hypothesis holds the most relevance. However, there is still not enough information available regarding the exact role exhibited by Aβ peptides in the body [13].

The review provides an extensive elaboration of the therapeutic candidates targeting amyloid peptides in AD. The amyloid cascade hypothesis is considered to be the most relevant and accepted form of AD hypothesis, which has acquired the interest of various researchers to investigate more about its role in AD. The authors discuss the possible AD treatment therapies, such as β- and γ-secretase modulators, which retard the enzymatic processing of APP, thus hindering the formation of Aβ monomers; active and passive immunotherapeutic regimes, where the former involves cellular and humoral immune response generation via T and B cell activation and the latter accounts for the administration of antibodies targeting Aβ plaques; the removal of amyloid aggregates, either by the activation of enzymatic pathways (the ubiquitin–proteasome pathway and lysosome-induced degradation) or modulating the Aβ circulation between the brain and peripheral blood circulation; peptide inhibitors of amyloid aggregation; bapineuzumab-mediated changes in vascular permeability; reduction in the accumulation and neurotoxicity of Aβ by GLP-1; statin-mediated Aβ peptide degradation; ibuprofen (NSAID)-mediated inhibition of nicotinamide adenine dinucleotide phosphate oxidase activation and ROS production; and natural drugs and dyes (methylene blue, EGCG, orcein, silibinin, Congo red, proline, NeuroDefend and so on). All the therapeutic possibilities targeting Aβ peptides are highlighted in the text, evidently supported by appropriate investigations and studies. Therefore, the review aims to create a clear picture of treatment therapies targeting the amyloid cascade and provide a significant opportunity to the neurologists and researchers all over the globe to study and evaluate the treatment paradigm and exact cause behind the deteriorated neuronal functions in AD.

## Figures and Tables

**Table 1:** Therapeutic agents targeting Aβ peptides in Alzheimer’s disease (AD).

Therapeutic Agents Targeting Amyloidogenic Events	Action	Ref.
Novel beta-secretase inhibitor (KMI-429) with hydroxy-methyl-carbonyl (HMC) isostere	Beta-site APP-cleaving enzyme (BACE1) blockers	[52]
N-benzoyl-oxy-carbonyl-valine-leucine-leucinal (Z-VLL-CHO, C_25_H_39_N_3_O_5_)	β-secretase inhibitor, BACE1 blocker	[2]
Beta-secretase inhibitor GRL-8234	β-secretase inhibitor	[54,55,56,57]
Iso-liquiritigenin	BACE1 inhibitor	[59]
(*R*)-6-[(1,1′-biphenyl)-4-ylmethoxy]-1,2,3,4-tetrahydro-*N*,*N*-dimethyl-2-naphthalene-ethan-amine hydrochloride monohydrate (TAK-070, C_27_H_31_NO)	BACE1 inhibitor	[54]
BIIB042	γ-secretase modulator	[63]
NGP328 and NGP555	γ-secretase modulator	[64]
SGSM-36	γ-secretase modulator	[67]
LY450139	γ-secretase inhibitor	[54]
Aβ fragments in conjugation with poly-lysine	Active immunotherapeutic agents	[2]
NAB61	Passive immunotherapeutic agent	[78]
Anti-beta-amyloid monoclonal antibody (BAM-10)	Passive immunotherapeutic agent	[81]
Neprilysin (NEP)	Aβ load-reducing enzyme	[2]
Endothelin-converting enzyme (ECE)	Aβ load-reducing enzyme	[84]
Protein kinase C epsilon (PKCε)	ECE enhancer, Aβ load reducer	[85]
Serine protease plasmin	Amyloid degradation	[87]
Matrix metalloproteinases (MMPs)	Amyloid degradation	[88,89,90]
Estrogen	MMP-2, -9 enhancer, Aβ clearance	[91]
ATP-binding cassette (ABC) transporters	Prevent Aβ accumulation	[2]
Low-density lipoprotein receptor-related protein 1 (LRP1)	Prevent Aβ accumulation	[2]
Aβ16-20	Aβ-aggregation inhibitor	[94]
Aβ15-25	Aβ-aggregation inhibitor	[13]
OR-2	Aβ-aggregation inhibitor	[97]
RI-OR2	Aβ-aggregation inhibitor	[98]
Aggregated human beta-amyloidAN1792	Active immunotherapeutic reagent	[71]
Bapineuzumab	Monoclonal antibody	[54]
Glucagon-like peptide (GLP-1)	Aβ accumulation inhibitor	[115]
Statins	Aβ degradation promoter	[54]
Non-steroidal anti-inflammatory drugs (NSAIDs) (i.e., ibuprofen)	Curbed Aβ levels and ROS mitigation, γ-secretase modulator	[119,120,121]
Bismuth oxychloride (BiOCl) nanosheets	Destabilization of β-sheets	[123]
Congo red	Amyloid-inhibiting dye	[124,125]
Methylene blue	Amyloid-inhibiting dye	[126,127]
Thioflavin T	Amyloid-inhibiting dye	[128]
Orcein	Amyloid-inhibiting dye	[129]
Curcumin	Amyloid-inhibiting dye	[130]
Epi-gallocatechin-3-gallate (EGCG)	Aβ fibrillization inhibitor	[133]
Silibinin	Aβ aggregation inhibitor	[134]
Proline	β-sheet breaker	[135]
NeuroDefend	Aβ load reduction	[136]
Naturally obtained dietary flavonoids	CDK-5 and GSK-3β inhibitors, secretase enzyme modulators, Aβ aggregation inhibitors	[137,138]
Autacoid local injury antagonist amides (ALIAmides) (palmitoyl ethanol amide, PEA)	Anti-inflammatory, anti-hyperalgesia and lipid metabolism regulator	[107,110]
Ultra-micronized form of PEA and luteolin	Neuroinflammation amelioration	[107,108]
